# Factors associated with home death in South Korea: Using the exit data from the Korean Longitudinal Study of Aging, 2008–2018

**DOI:** 10.1371/journal.pone.0288165

**Published:** 2023-07-14

**Authors:** Jooyoung Cheon, Dong Hee Kim, Chung Min Cho

**Affiliations:** Department of Nursing Science, Sungshin Women’s University, Seoul, Republic of Korea; Sungkyunkwan University School of Social Sciences, REPUBLIC OF KOREA

## Abstract

**Background:**

Even though home deaths have been reported to improve quality of life, satisfy patients and families, and reduce healthcare expenditures, not enough is known about the factors that influence home deaths in Korea.

**Objectives:**

This study aimed to examine the factors associated with home deaths among middle-aged and older adults in South Korea.

**Methods:**

This secondary data analysis used core interview and exit interview data of the Korean Longitudinal Study of Aging conducted between 2008 and 2018. The deceased included adults over the age of 45 years. The exit data were obtained from interviews with family members or other acquaintances known to the deceased every two years since 2008. Complex-sample logistic regression was conducted using 1,565 middle-aged and older deceased adults.

**Results:**

Among 1,565 decedents, the average age at the time of death was 80.67±10.69 in the home death group, and 78.72±9.83 in the non-home death group. The proportion of home-related deaths was 26.4%. Age over 81 years was associated with increased odds of home death, whereas having two or more living children, living in town/small city, paid medical expenses by children/grandchildren and their spouses, expected death, death from disease, and having three or more chronic diseases were associated with decreased odds of home death. An increase in activities of daily living during three months before death was associated with a decrease in home death.

**Conclusion:**

The findings could help healthcare professionals develop tailored interventions to help people die at their preferred place of death based on family characteristics and healthcare accessibility. Age, residential area, number of children and children’s financial support, and illness-related factors influenced home death by creating differences in access to healthcare resources and support. Policymakers should decrease healthcare disparities and improve health resource allocation and home-based care.

## Introduction

Due to increases in life expectancy and population aging, identifying end-of-life preferences has become more important and is one of the biggest concerns for healthcare policymakers [1]. In particular, the preference for place of death is a key indicator of palliative care quality [2,3]. Similarly, home death is sometimes considered a potential indicator of end-of-life care quality [2–5]. Previous studies have reported that home death improves the quality of dying, satisfies patients and family members, and reduces healthcare expenditures [1–3,6].

Unfortunately, many terminally ill patients do not receive the care they desire, in the place they want to stay in, and at the right time [4,5,7,8]. Often, patients neither know their preferred place of death in advance nor do they discuss it with their families until they become seriously ill. A lack of awareness results in patients’ death before they even think about dying place preferences or even discuss it with their families and healthcare providers [3,9].

Due to cultural differences, people from Eastern cultures are more likely to avoid expressing their end-of-life treatment preferences or having end-of-life discussions with their family members than are people from Western cultures [10–12]. Moreover, in Eastern–and especially Confucian–culture, the family’s decisions are given preference over patients’ decisions. This is based on the principle of autonomy in Western culture and occurs because families act as a support system, and the concept of filial piety may influence the final decisions among adult children of older adults [10–12]. Meanwhile, some older adult patients allow their children to make health decisions on their behalf since they assume their children to be more knowledgeable [11]. Therefore, it is important to consider cultural aspects in the end-of-life decision-making process because older adults from Eastern cultures value family decisions or their children’s decisions more than older adults from Western cultures do.

In Korea, more than half the patients have died in hospitals in the last decade, and this proportion is increasing. Most Koreans prefer to receive terminal treatment or die at home, especially as they age [1]. However, the 2020 South Korean mortality statistics reported 75.6% of deaths at medical institutions, and the death rate at medical institutions was higher than that at homes. The institutional death rate by age are as follows: 15–29 years old (42.3%), 30–44 years old (52.1%), 45–64 years old (70.8%), 65–84 years old (80.6%), and 85 years and above (73.9%). This indicates that the rate of death in medical institutions increases sharply from the age of 45 or older [13]. This is because there are higher chances of people getting chronic diseases as they age; thus, older adults require more hospital care and are more likely to die in hospitals where they are treated than healthier young people [13].

In the case of ‘Grandma Kim’ in 2009 in South Korea, the Supreme Court ruled that “meaningless life-sustaining treatment can be stopped,” which has led to active social discussions regarding terminal treatment and the quality of life before death. The “Law on Hospice and Palliative Care and Determination of Life-Sustaining Treatment for Terminally Ill Patients” has been in force since February 2018, with growing social concerns about dignified and comfortable end-of-life or death [14]. However, factors related to home death in South Korea are not well known despite the increase in social interest and law enactment.

Previous studies found that factors associated with home death were age, gender, marital status, socioeconomic status, residence (urbanization), comorbidity, symptom distress, poorer self-rated health, family-related factors, caregiver burden, healthcare accessibility, and the healthcare system [1,3,5,15,16]. Identifying the factors influencing home death among middle-aged and older adults could help healthcare professionals provide appropriate interventions based on their background. Policymakers could also develop policies to promote home death when adults prefer to die at home as a component of good death [2,17].

Using death data from the Korean Longitudinal Study of Aging (KLoSA), an ongoing longitudinal panel survey, this study aimed to determine factors related to death at home among middle-aged and older adults in South Korea. Regarding healthcare utilization [18], we examined the effect of predisposing factors to disease, enabling resources, and the need for health services in home deaths. The study findings could provide insight into home deaths and help healthcare policymakers improve healthcare utilization.

## Materials and methods

### Study design and data source

This cross-sectional and correlational study performed a secondary analysis of data from the KLoSA conducted by the Korea Labor Institute and Korea Employment Information Service. The KLoSA is an ongoing longitudinal panel survey designed to be representative of the South Korean population aged above 45 years [19]. It is also advantageous to examine the relationship between middle- and older-aged life and health based on Korea’s tendency to retire from the mid-40s onwards after the economic crisis in the late 1990s [19]. In 2006, 10,254 community-dwelling adults (born in 1961 or earlier) were randomly selected using a multistage and stratified sampling method based on geographical areas and housing types across Korea. Middle-aged and older adults have been interviewed every two years since 2006 using computer-assisted personal interviewing. The seventh wave (core interview data + exit interview data) was completed for 9,510 in 2018 and showed a retention rate of 78.8%, with 7,491 (core interview data of 6,940 + exit interview data of 551) valid samples available for analysis [20]. The cumulative number of uninvestigated deaths was 139 in the seventh wave [20].

The core interview data included information on household background, human attributes, family, health, socioeconomic status, subjective expectations, and quality of life. If the respondent was unable to communicate due to advanced age or was unable to respond directly due to illness, the KLoSA allowed family members or other acquaintances to help. Family members or other acquaintances were spouse, parents, parents in law, siblings, spouse’s siblings, children, children’s spouses, grandchildren, other relatives, and friends. When conducting proxy responses, questions were included for each survey area to confirm the degree of proxy response and the relationship with the proxy respondent [21]. When the death of the respondent was confirmed, the KLoSA conducted exit interviews with family members or other acquaintances of the deceased respondents. The survey was conducted using computer-assisted personal interviewing after obtaining informed consent, and an incentive was provided upon the completion of the survey.

**Fig 1:**
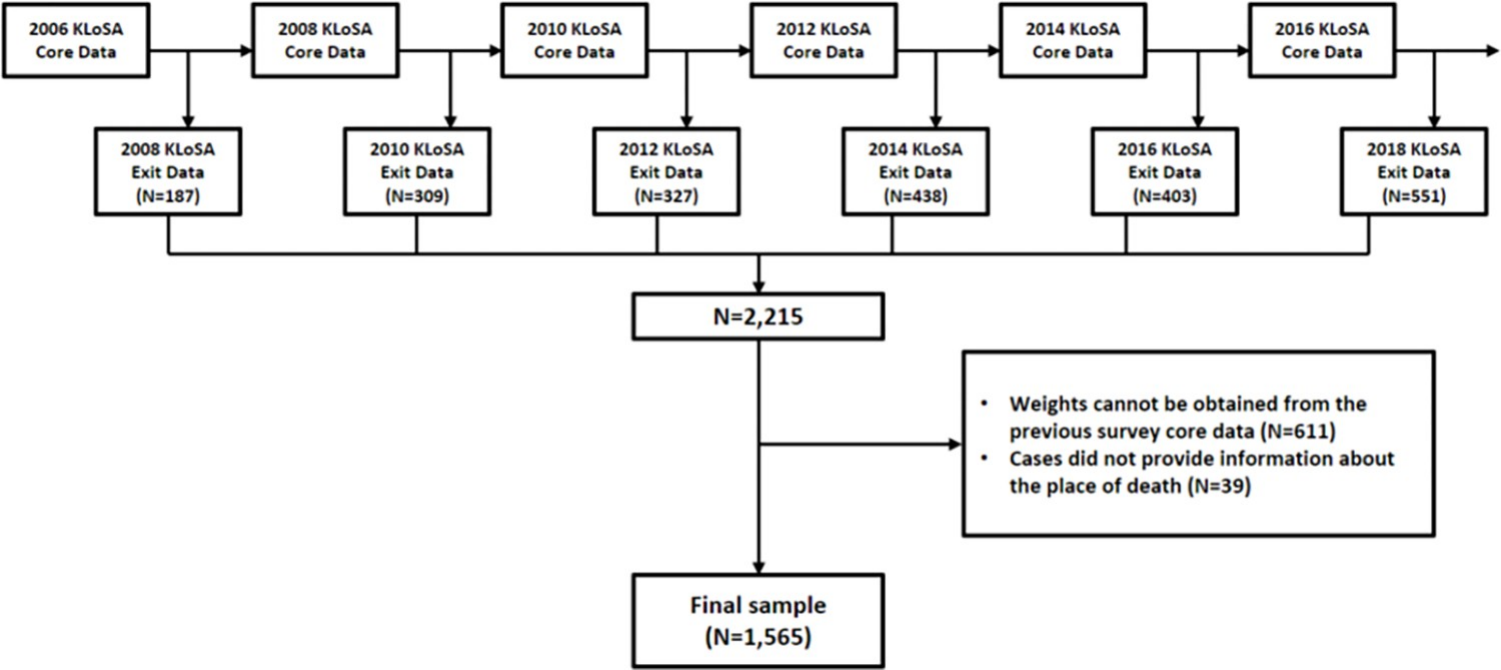
Sampling process.

The exit interview data included death-related information such as year, place, cause, pre-death health condition, and other pre-death situations. This study used core interview data from the first wave (2006) through the sixth wave (2016) and exit interview data from the second wave (2008) through the seventh wave (2018) (Fig 1). Detailed information was obtained from the KLoSA website (https://survey.keis.or.kr/eng/klosa/klosa01.jsp).

#### Participants

All deceased individuals aged 45 and above were selected from the exit interview data between 2008 and 2018. Of the 2,215 deceased, we excluded data points without information on 1) weight from previous core interview data (N = 611) and 2) place of death (n = 39). Finally, 1,565 were included in the analysis (Fig 1).

#### Variables

This study adopted Andersen’s healthcare utilization model which has been used extensively in studies examining healthcare use [18,22]. This model reflects the multiple influences on health services’ use by including numerous iterations and feedback loops that represent the mutual influence of an individual’s characteristics, predisposing factors, perceived need and health behavior [18,22].It has been used to examine the multiple influences of population characteristics, including predisposing characteristics (the likelihood that individuals will need health services); enabling resources (individuals’ ability to access services); and the need for health services (perceived and evaluated needs) on health behavior (personal health practices and use of health services) and health status outcomes (perceived health status, evaluated health status, and consumer satisfaction). The variables in this study were selected based on evidence from previous studies regarding place of death and on the health care utilization model among variables provided by the KLoSA dataset [1,3,5,15,16,18].

In this study, predisposing characteristics included age at the time of death, gender, marital status, number of living children, and residential area. Enabling resources included the main bearer of medical expenses. The need for health services included expected death, cause of death, total number of chronic diseases (0–11; stroke, hypertension, diabetes, cancer, lung disease, heart disease, liver disease, dementia or memory disturbance, mental disorders such as depression, fracture or aftermath of traffic accident, and osteoarthritis), cognitive impairment before death, pain experienced during the year before death, and activities of daily living (ADL) during the last three months before death (0–7; dressing, washing one’s face, bathing/showering, eating, leaving the room after waking up, using the toilet, and controlling urination/defecation). Variables for health behaviors were not selected in this study because it could not be determined if proxy can represent the decedent’s personal health practices before death. Also, variables of use of health services, such as hospital visits, intensive care unit use, and medication use, had too many missing values to be included as variables. The health outcome was the place of death. The place of death was categorized into home, hospital, and assisted living facilities (nursing home/senior facilities). We recorded the variables as home and non-home deaths.

#### Statistical analyses

Descriptive analyses were conducted using mean, standard deviation, number, and percentage (%). Independent t-tests and chi-square tests were used to identify important factors. We examined the correlation between independent variables to avoid collinearity. To analyze the association between the variables, complex-sample logistic regression was conducted. The KLoSA has a multistage stratified design; therefore, both cross-sectional weights and longitudinal weights are provided in the final analyses to correct for the differential probability of sample selection. As this study was a cross-sectional study using exit data, the researchers used cross-sectional weights for the final analysis. The outcomes are presented as odd ratios and 95% confidence intervals (CIs). All statistical analyses were performed using the IBM SPSS statistical package (IBM Corp., Armonk, NY, USA). A p-value of .05 was considered statistically significant.

#### Ethics

The KLoSA survey was approved by the state in accordance with Article 18 of the Statistics Act (approval number 33602) and was conducted after obtaining the informed consent of the study participants [23]. The responses were treated with the utmost confidentiality in accordance with the Statistics Act (Articles 33 and 34) and were not used for anything other than statistical purposes. The data, which have been anonymized, are available to the public on the survey website and can be downloaded. The researchers obtained permission to use public data and downloaded the data from the KLoSA website. The study conducted secondary data analysis using publicly available data. Therefore, this study did not harm the participants, and anonymity and confidentiality were guaranteed. This study was exempt from institutional review board approval of Sungshin Women’s University (SSWUIRB-2021-035) because it was a secondary analysis of published data and no new patients were involved in this study.

## Results

### Characteristics of the sample

Among 1,565 decedents, the average age at the time of death was 79.24±10.10 overall, 80.67±10.69 in the home death group, and 78.72±9.83 in the non-home death group. In Table 1, most decedents were aged 81 years or older (49.3%), and half (53.2%) were men. Most were married (58.2%) and had no religion (61.0%). Over half of them had more than four children. The majority of the home death group lived in rural areas (40.0%), whereas a major portion of the non-home death group lived in a large city (38.2%).

**Table 1:** Characteristics of the decedents (N = 1,565).

Variables	Total	Home deaths (N = 417; 26.6%)	Non-home deaths (N = 1148; 73.4%)
	N(%)		
**Age at the time of death**			
** 45–65**	157 (10.1)	43 (10.3)	114 (10.0)
** 66–80**	633 (40.7)	139 (33.4)	494 (43.3)
** 81≤**	767 (49.3)	234 (56.3)	533 (46.7)
**Gender**			
** Women**	732 (46.8)	207 (49.6)	525 (45.7)
** Men**	833 (53.2)	210 (50.4)	623 (54.3)
**Marital status**			
** Not married**	615 (39.3)	192 (47.4)	423 (37.7)
** Married**	911 (58.2)	213 (52.6)	698 (62.3)
**Number of living children**			
** 0–1**	128 (8.2)	46 (11.1)	82 (7.2)
** 2–3**	598 (38.3)	144 (34.6)	454 (39.6)
** 4≤**	836 (53.5)	226 (53.5)	610 (53.2)
**Residential area**			
** Rural**	512 (32.7)	167 (40.0)	345 (30.1)
** Town/Small city**	461 (29.5)	97 (23.3)	364 (31.7)
** Large city**	592 (37.8)	153 (36.7)	439 (38.2)
**Main bearer of medical expenses**			
** Decedent**	301 (19.8)	101 (24.8)	200 (17.9)
** Spouse**	223 (14.7)	60 (14.7)	163 (14.6)
** Children/Grandchildren and their spouses**	998 (65.6)	246 (60.4)	752 (67.4)
**Expected death**			
** Unexpected sudden death**	411 (26.3)	157 (37.6)	254 (22.1)
** Somewhat expected, but sudden death**	632 (40.4)	166 (39.8)	466 (40.6)
** Expected death**	521 (33.3)	94 (22.5)	427 (37.2)
**Cause of death**			
** Others (violent/accidental death, unknown)**	232 (14.8)	105 (25.2)	127 (11.1)
** Death from disease**	1332 (85.2)	311 (74.8)	1021 (88.9)
**Total number of chronic diseases**			
** 0–1**	1123 (72.0)	327 (78.6)	796 (69.6)
** 2**	266 (17.1)	63 (15.1)	203 (17.8)
** 3≤**	170 (10.9)	26 (6.3)	144 (12.6)
**Cognitive impairment before death**			
** Yes**	491 (31.4)	111 (26.6)	380 (33.1)
** No**	1073 (68.6)	305 (73.4)	767 (66.9)
**Pain experienced during the year before death**			
** Yes**	558 (35.7)	115 (27.6)	443 (38.7)
** No**	1005 (64.3)	302 (72.4)	703 (61.3)
**ADL during three months before death (range: 0–7) (Mean±SD)**	4.19±3.18	3.35±3.23	4.50±3.11

Note. Unweighted results; missing data were excluded from analysis; SD = standard deviation; ADL = activities of daily living; chronic diseases included stroke, hypertension, diabetes, cancer, lung disease, heart disease, liver disease, dementia or memory disturbance, mental disorders such as depression, fracture or aftermath of traffic accident, and osteoarthritis.

The main bearers of medical expenses were children, grandchildren, and their spouses (65.6%). One-third of the deaths were expected (33.3%), and most deaths were caused by disease (85.2%). The average number of chronic diseases was 1.38±0.89, and most decedents (72.0%) had 0–1 chronic disease. A few decedents had cognitive impairment before death (31.5%), and around one-third experienced pain experienced during the year before death (35.7%). The average ADL during the three months before death was 4.19±3.18 in total, 3.35±3.23 in the home death group, and 4.50±3.11 in the non-home death group.

#### Bivariate results

As shown in Table 2, 26.4% died at home. In terms of demographic characteristics and socioeconomic status, there were significant differences in age (χ^2^ = 14.628, *p* = .011), marital status (χ^2^ = 9.996, *p* = .004), residential area (χ^2^ = 18.265, *p* = .001), and main pay for medical expenses (χ^2^ = 15.687, *p* = .003). Among the death-related factors, there were significant differences in expected death (χ^2^ = 51.736, *p* < .001), cause of death (χ^2^ = 52.124, *p* < .001), total number of chronic diseases (χ^2^ = 15.412, *p* = .002), cognitive impairment before death (χ^2^ = 7.924, *p* = .008), pain experienced during the year before death (χ^2^ = 16.615, *p* < .001), and ADL during the months before death (χ^2^ = 42.465, *p* < .001).

**Table 2:** Home deaths according to the characteristics of the decedents (N = 1,565).

Variables	Total	Home deaths (26.4%)	Non-home deaths (73.6%)	t or χ^2^ (p-value)
	**%**			
**Age at the time of death**				14.628 (.011) *
** 45–65**	18.3	19.7	17.7	
** 66–80**	40.0	32.1	42.8	
** 81≤**	41.8	48.2	39.5	
**Gender**				3.365 (.104)
** Women**	46.5	50.4	45.2	
** Men**	53.5	49.6	54.8	
**Marital status**				9.996 (.004) *
** Not married**	39.5	46.2	37.2	
** Married**	60.5	53.8	62.8	
**Number of living children**				7.054 (.084)
** 0–1**	10.0	13.3	8.8	
** 2–3**	43.0	40.6	43.8	
** 4≤**	47.1	46.1	47.4	
**Residential area**				18.265 (.001) *
** Rural**	32.8	40.3	30.1	
** Town/Small city**	31.1	24.1	33.6	
** Large city**	36.1	35.6	36.3	
**Main bearer of medical expenses**				15.687 (.003) *
** Decedent**	20.9	27.8	18.5	
** Spouse**	16.3	15.4	16.6	
** Children/Grandchildren and their spouses**	62.8	56.8	65.0	
**Expected death**				51.736 (< .001) *
** Unexpected sudden death**	28.7	40.5	24.5	
** Somewhat expected, but sudden death**	39.8	39.7	39.9	
** Expected death**	31.5	19.8	35.7	
**Cause of death**				52.124 (< .001) *
** Others (violent/accidental death, unknown)**	15.3	26.3	11.4	
** Death from disease**	84.7	73.7	88.6	
**Total number of chronic diseases**				15.412 (.002) *
** 0–1**	72.2	17.0	10.8	
** 2**	17.0	15.4	17.5	
** 3≤**	10.8	6.1	12.5	
**Cognitive impairment before death**				7.924 (.008) *
** Yes**	28.9	23.5	30.8	
** No**	71.1	76.5	69.2	
**Pain experienced during the year before death**				16.615 (< .001) *
** Yes**	36.7	28.4	39.7	
** No**	63.3	71.6	60.3	
**ADL during three months before death (Mean±SE)**	4.04±0.94	3.16±0.18	4.36±0.11	42.465 (< .001) *

Note. Weighted results

*p < 0.05; SE: Standard error; ADL = activities of daily living.

#### Logistic regression results

The results of the multivariate logistic regression analysis are shown in Table 3. In the model, the significant factors were age at the time of death, number of living children, residential area, main bearer of medical expenses, expected death, cause of death, total number of chronic diseases, and ADL during the three months before death. Gender, marital status, cognitive impairment before death, and pain experienced during the year before death were not significant factors. The model explained 10.6% (Cox and Snell R^2^) and 15.6% (Nagelkerke R^2^) of the variance in the odds of home deaths. The model fit was satisfactory and significant (F = 6.765, *p* < .001).

**Table 3:** Factors associated with home death (N = 1,565).

Variables	AOR	95% CI
**Age at the time of death**		
** 45–65**	1 (Reference)	
** 66–80**	0.050	0.621–1.777
** 81≤**	1.935	1.107–3.384*
**Gender**		
** Women**	1 (Reference)	
** Men**	0.962	0.697–1.328
**Marital status**		
** Not married**	1 (Reference)	
** Married**	0.785	0.559–1.101
**Number of lived children**		
** 0–1**	1 (Reference)	
** 2–3**	0.499	0.294–0.850*
** 4≤**	0.500	0.300–0.832*
**Residential area**		
** Rural**	1 (Reference)	
** Town/Small city**	0.525	0.371–0.744*
** Large city**	0.741	0.539–1.018
**Main bearer of medical expenses**		
** Decedent**	1 (Reference)	
** Spouse**	1.093	0.688–1.736
** Children/Grandchildren and their spouses**	0.680	0.476–0.972*
**Expected death**		
** Unexpected sudden death**	1 (Reference)	
** Somewhat expected, but sudden death**	0.922	0.634–1.340
** Expected death**	0.497	0.328–0.754*
**Cause of death**		
** Others (violent/accidental death, unknown)**	1 (Reference)	
** Death from disease**	0.432	0.295–0.633*
**Total number of chronic diseases**		
** 0–1**	1 (Reference)	
** 2**	0.926	0.631–1.360
** 3≤**	0.443	0.265–0.739*
**Cognitive impairment before death**		
** Yes**	1 (Reference)	
** No**	0.966	0.03–1.328
**Pain experienced during the year before death**		
** Yes**	1 (Reference)	
** No**	0.961	0.701–1.318
**ADL during three months before death**	0.940	0.895–0.987*
	Cox & Snell = .106, Nagelkerke = .156 Wald F = 6.765, p < .001	

Note. AOR = adjusted odds ratio; CI = confidence interval

*p < 0.05; ADL = activities of daily living.

Decedents aged ≥ 81 years were more likely to die at home than those aged 45–65 years [adjusted odds ratio (AOR) = 1.935, 95% confidence interval (CI) = 1.107–3.384]. The odds of home death for decedents aged 66–80 were not significantly different than those for decedents aged 45–65 years (AOR = 0.050, 95% CI = 0.621–1.777). Compared to decedents who had 0–1 number of living children, the odds of home death were lower for decedents having living children of 2–3 (AOR = 0.499, 95% CI = 0.294–0.850) and 4 or more (AOR 0.500, 95% CI = 0.300–0.832). The odds of home death in town/small city residents were lower than those in rural areas (AOR = 0.525, 95% CI = 0.371–0.744), while the odds of home death in large city were not significantly different in rural areas (AOR = 0.741, 95% CI = 0.539–1.018). Decedents whose medical expenses were borne by children/grandchildren and their spouses were less likely to die at home than those with self-paid medical expenses (AOR = 0.680, 95% CI = 0.476–0.972). The odds of home death for decedents whose medical expenses were borne by their spouse were not significantly different for those with self-paid medical expenses (AOR = 1.093, 95% CI = 0.688–1.736). Expected death was significantly associated with lower odds of home death than unexpected sudden death (AOR = 0.497, 95% CI = 0.328–0.754), while the odds of somewhat expected, but sudden home death were not significantly different from unexpected sudden home death (AOR = 0.922, 95% CI = 0.634–1.340). Decedents who died of disease were associated with lower odds of home death than those who died from violent/accidental events or unknown causes (AOR = 0.432, 95% CI = 0.295–0.633). The odds of home death for decedents with three or more chronic diseases were 0.443 times the odds of those with number 0–1 (AOR = 0.443, 95% CI = 0.265–0.739) while the odds of home for decedents with two chronic diseases were not significantly different from the odds of those with number 0–1 (AOR = 0.926, 95% CI = 0.631–1.360). The odds of home death decreased with an increase in ADL during three months before death (AOR = 0.940, 95% CI = 0.895–0.987).

## Discussion

Although home death has been considered a component of a good death, many people still die at hospitals and long-term care facilities [1,2,4,13]. In the aspect of health care service use based on the Andersen’s healthcare utilization model, this study examined factors associated with death at home in Korea using a longitudinal panel survey designed to represent adults aged 45 and above. Home death as a health outcome was affected by predisposing characteristics (age at the time of death, number of living children, residential area), enabling factors (main bearer of medical expenses), need for health services (expected death, cause of death, total number of chronic diseases, and ADL during the three months before death).

In this study, the rate of non-home deaths was 73.6%, which was higher than the rate of 38.6% in hospital and hospice facilities in the USA [6]. In the past few decades, rapid socio-economic changes such as urbanization, lifestyle, and household structure have made it impossible to maintain the traditional family support system for patients in South Korea. Therefore, many patients needing assistance at the end of life must be admitted to hospitals or facilities to receive appropriate care, resulting in increased hospital mortality rates [1,9].

The residential area was a significant factor influencing home deaths in South Korea. Home deaths were higher in rural areas than in towns and/or small cities. Previous studies reported a higher death rate in city hospitals (or urban areas) [1,15,24]. In general, the proportion of older people is higher in rural areas than in cities, which indicates that the average age in rural areas is higher than that in cities in Korea [1]. This may be related to the increase in the number of home deaths. Health disparities also exist in deaths at home [5,25]. Patients living in cities have better access to treatment and healthcare support systems [1]. In Seoul, South Korea, the concentration of healthcare professionals, such as doctors and nurses, is increasing [26]. Low hospital density in low-income municipalities is associated with high home mortality rates [5]. The difference in age group according to residential area and the difference in accessibility to healthcare resources and support affected the rate of home death. Therefore, health policymakers should consider these characteristics when allocating medical resources based on healthcare utilization.

A higher number of children may be related to an increase in payment capability for formal healthcare, which leads to a reduced financial burden [12]. This finding contradicts the previous systematic review finding that a high rate of home death was associated with a positive financial situation [24]. This is due to differences between the Western and Korean cultures. Specifically, in Eastern culture, older adults have strong expectations of children’s support and decision-making regarding their end-of-life care, and they often allow their children to make health decisions on their behalf [10,11]. Therefore, as the number of children increases, hospitalization is easy even with relatively low finances [1], which can influence children’s decision to hospitalize their parents.

Interestingly, decedents with a higher number of children and whose principal medical expense bearers were children or grandchildren were less likely to die at home, which is consistent with previous findings [10,12,16]. Many adult children still think that doing their best is filial piety [12]. In Confucian culture, children are the main source of support and care for their parents and are one of the main decision-makers at the end of life [10,12]. Children may think that hospital treatment is the best option for critically ill parents who suffer from pain and symptoms at the end of life.

People whose death is expected die in hospitals rather than at home, as shown in this study’s findings. Many expected deaths are caused by chronic diseases, and most unexpected deaths are caused by various incidents, including traffic accidents, addiction, falls, drowning, suicide, and murder [19]. Unexpected sudden death does not provide an opportunity for patients to receive hospital treatments. Many patients whose death is expected are admitted to hospitals or facilities to receive palliative care to relieve pain and symptoms [1,9].

The prognosis, disease severity, and patient’s ability to perform ADL are related to at-home mortality [1,3,11]. Although most people believe that home care is necessary for a peaceful end-of-life experience [5], most terminally ill patients usually die while receiving treatment in hospitals [1]. Even patients at advanced stages of the disease may die in the hospital due to wider treatment options, despite knowing more about the prognosis [2]. Specifically, patients with more chronic diseases or poorer self-rated health preferred medical facilities to home as their place of death [2,3].

Further studies are needed to continuously follow the change in preferences for the place of death and the actual place of death because the law for hospice and terminally ill patients came into effect in 2018; therefore, it is unknown whether the changes after the law were applied in South Korea. A previous study reported that half of the patients had no preferred place of death at hospital admission, and they were often unaware of their preferences regarding the place of death before someone began to discuss this topic [3]. Receiving terminal care at home and documenting preference for a place of death increased the odds of at-home death; however, hospital admission for an acute problem at the time of death was associated with a lower rate of at-home mortality [12]. These findings emphasize the importance of healthcare professionals providing patients and families with appropriate information regarding diagnosis and prognosis and discussing end-of-life treatment at an early stage. Healthcare professionals can provide an opportunity for terminally ill patients and their family caregivers to think about and discuss their preferred place of treatment and death [1–3,10,27].

This study had several limitations that warrant consideration when interpreting the findings. First, the exit data relied on proxy responses after the respondent had died, which may lead to concerns about the recall bias. Ideally, when the data are obtained from proxies soon after patients’ deaths, the internal validity and the reliability of data can be improved. Second, even though this study included six waves of exit data between 2008 and 2018, we could not include all eligible deceased individuals for several reasons, which resulted in selection bias. Finally, home death does not always indicate a good death because it may often include sudden death, suicide, and accidental death. Therefore, further research is warranted to examine the factors influencing home deaths according to the cause of death.

## Conclusion

Dying at the preferred place of death is an important end-of-life issue. However, there has been an increase in the death rate at hospitals and long-term care facilities, despite people desiring death at home in South Korea. In this study, residential area, the number of children and children’s financial support, and the predictability of deaths and severity of diseases influenced home death by creating differences in access to healthcare resources and support. The findings regarding factors influencing home death in this study could help healthcare professionals develop tailored interventions based on family characteristics, healthcare accessibility, and utilization. Additionally, healthcare policymakers should make efforts to decrease healthcare disparities, improve resource allocation, and foster formal caregivers for home-based care.

## References

[1] Mai TT, Lee E, Cho H, Chang YJ. Increasing trend in hospital deaths consistent among older decedents in Korea: A population-based study using death registration database, 2001–2014. BMC Palliat Care. 2018;17: 16. doi: 10.1186/s12904-017-0269-x 29325534 PMC5765638

[2] Fereidouni A, Rassouli M, Salesi M, Ashrafizadeh H, Vahedian-Azimi A, Barasteh S. Preferred place of death in adult cancer patients: A systematic review and meta-analysis. Front Psychol. 2021;12: 704590. doi: 10.3389/fpsyg.2021.704590 34512460 PMC8429937

[3] van Doorne I, van Rijn M, Dofferhoff SM, Willems DL, Buurman BM. Patients’ preferred place of death: Patients are willing to consider their preferences, but someone has to ask them. Age Ageing. 2021;50: 2004–2011. doi: 10.1093/ageing/afab176 34473834 PMC8581384

[4] García-Sanjuán S, Fernández-Alcántara M, Clement-Carbonell V, Campos-Calderón CP, Orts-Beneito N, f-Martínez MJ. Levels and determinants of place-of-death congruence in palliative patients: A systematic review. Front Psychol. 2021;12: 807869. doi: 10.3389/fpsyg.2021.807869 35095694 PMC8792401

[5] Ikeda T, Tsuboya T. Place of death and density of homecare resources: A nationwide study in Japan. Ann Geriatr Med Res. 2021;25: 25–32. doi: 10.4235/agmr.21.0003 33794586 PMC8024167

[6] Chino F, Kamal AH, Leblanc TW, Zafar SY, Suneja G, Chino JP. Place of death for patients with cancer in the United States, 1999 through 2015: Racial, age, and geographic disparities. Cancer. 2018;124: 4408–4419. doi: 10.1002/cncr.31737 30343501

[7] Hyun MK, Jung KH, Yun YH, Kim YA, Lee WJ, Do YR, et al. Factors associated with place of death in Korean patients with terminal cancer. Asian Pac J Cancer Prev. 2013;14: 7309–7314. doi: 10.7314/APJCP.2013.14.12.7309 24460293

[8] National Agency for Management of Life-Sustaining Treatment. Life-sustaining treatment information portal, monthly statistics; 2021. [cited November 1, 2021]. Available from: https://www.lst.go.kr/comm/monthlyStatistics.do.

[9] Kim SH, Kang S, Song MK. Intensity of care at the end of life among older adults in Korea. J Palliat Care. 2018;33: 47–52. doi: 10.1177/0825859718754398 29361883

[10] Cheon J. Completion of advance directive and withdrawal of life-sustaining treatments among older adults in South Korea: A systematic review. ksles. 2020;27: 473–488. doi: 10.21086/ksles.2020.08.27.4.473

[11] Dutta O, Lall P, Patinadan PV, Car J, Low CK, Tan WS, et al. Patient autonomy and participation in end-of-life decision-making: an interpretive-systemic focus group study on perspectives of Asian healthcare professionals. Palliat Support Care. 2020;18(4): 425–430. doi: 10.1017/S1478951519000865 31699170

[12] Tan WS, Bajpai R, Low CK, Ho AHY, Wu HY, Car J. Individual, clinical and system factors associated with the place of death: A linked national database study. PLOS ONE. 2019;14: e0215566. doi: 10.1371/journal.pone.0215566 30998764 PMC6472886

[13] Korea Statistics. Preliminary results of birth and death statistics in 2020; 2021 [cited 2021 Nov 1]. Available from: https://kostat.go.kr/board.es?mid=a20108100000&bid=11773&act=view&list_no=388763&tag=&nPage=1&ref_bid.

[14] Korea Institute for Health and Social Affairs. Announcement; 2019 [cited 2021 Nov 1]. Available from: https://www.mohw.go.kr/react/al/sal0301vw.jsp?PAR_MENU_ID=04&MENU_ID=0403&CONT_SEQ=349863&page=1.

[15] Cabañero-Martínez MJ, Nolasco A, Melchor I, Fernández-Alcántara M, Cabrero-García J. Place of death and associated factors: A population-based study using death certificate data. Eur J Public Health. 2019;29: 608–615. doi: 10.1093/eurpub/cky267 30601984

[16] Lei L, Gerlach LB, Powell VD, Maust DT. Caregiver support and place of death among older adults. J Am Geriatr Soc. 2021;69: 1221–1230. doi: 10.1111/jgs.17055 33590479 PMC8127348

[17] Ali M, Capel M, Jones G, Gazi T. The importance of identifying preferred place of death. BMJ Support Palliat Care. 2019;9: 84–91. doi: 10.1136/bmjspcare-2015-000878 26408428

[18] Andersen RM. Revisiting the behavioral model and access to medical care: Does it matter? J Health Soc Behav. 1995;36: 1–10. 7738325

[19] Survey Employment. KLoSA (Korean Longitudinal Study of Aging); 2021 [cited 2021 Nov 1]. Available from: https://survey.keis.or.kr/eng/klosa/klosa03.jsp

[20] Korea Employment Information Service. 2020 Korean Longitudinal Study of Aging (KLoSA) Basic Analysis Report. p. 6; 2021 [cited 2023 May 1]. Available from: https://www.keis.or.kr/user/extra/main/3873/publication/publicationList/jsp/LayOutPage.do?categoryidx=131&pubIdx=8537&spage=1.

[21] Statistics Korea. Periodic statistical quality assurance report: Korean Longitudinal Study of Aging (2016); 2017 [cited 2023 May 1]. Available from: https://kostat.go.kr/board.es?mid=a10409060100&bid=67&tag=&act=view&list_no=359027&ref_bid.

[22] Babitsch B, Gohl D, von Lengerke T. Re-revisiting Andersen’s Behavioral Model of Health Services Use: a systematic review of studies from 1998–2011. Psychosoc Med. 2012;9: Dec 11. doi: 10.3205/psm000089 23133505 PMC3488807

[23] Korea Employment Information Service. Announcements; 2018 [cited 2023 May 1]. Available from: https://survey.keis.or.kr/madang/notice/Read.jsp?ntt_id=4540.

[24] Neergaard MA, Brunoe AH, Skorstengaard MH, Nielsen MK. What socio-economic factors determine place of death for people with life-limiting illness? A systematic review and appraisal of methodological rigour. Palliat Med. 2019;33: 900–925. doi: 10.1177/0269216319847089 31187687

[25] Abe K, Taniguchi Y, Kawachi I, Watanabe T, Tamiya N. Municipal long-term care workforce supply and in-home deaths at the end of life: Panel data analysis with a fixed-effect model in Japan. Geriatr Gerontol Int. 2021;21: 712–717. doi: 10.1111/ggi.14200 34105232

[26] Korea Statistics. Korean social trends 2018; 2018 [cited 2021 Nov 1]. Available from: https://sri.kostat.go.kr/board.es?mid=a10301150000&bid=246&act=view&list_no=372006.

[27] Wahid AS, Sayma M, Jamshaid S, Kerwat D, Oyewole F, Saleh D, et al. Barriers and facilitators influencing death at home: A meta-ethnography. Palliat Med. 2018;32: 314–328. doi: 10.1177/0269216317713427 28604232

